# Anti-Inflammatory Effects of the Combined Treatment of Resveratrol- and Protopanaxadiol-Enriched Rice Seed Extract on Lipopolysaccharide-Stimulated RAW264.7 Cells

**DOI:** 10.3390/molecules29184343

**Published:** 2024-09-13

**Authors:** Chaiwat Monmai, So-Hyeon Baek

**Affiliations:** Department of Agriculture Life Science, Sunchon National University, Suncheon 59722, Republic of Korea; bbuayy@gmail.com

**Keywords:** cotreatment effect, resveratrol, protopanaxadiol, anti-inflammatory, NF-κB, MAPK, PGE_2_

## Abstract

The overproduction of proinflammatory cytokines triggers a variety of diseases. Protopanaxadiol (PPD) and resveratrol are naturally found in plants such as ginseng and have potential anti-inflammatory properties, and resveratrol- and PPD-enriched rice seeds have been previously successfully generated. Herein, the synergistic anti-inflammatory activities of extracts of these enriched seeds were assessed in lipopolysaccharide (LPS)-stimulated RAW264.7 cells. In comparison with treatment using extract prepared from PPD-producing transgenic rice (DJ-PPD) alone, cotreatment with DJ526 and DJ-PPD (TR_3) markedly enhanced the anti-inflammatory activities at a similar (compared to DJ526) or higher (compared to DJ-PPD) level. Cotreatment with DJ526 and DJ-PPD markedly inhibited the activation of nuclear factor kappa B (NF-κB) and mitogen-activated protein kinase (MAPK) signaling pathways. Thus, DJ526 and DJ-PPD in combination suppressed the expression of phosphorylated (p)-NF-κB p65, p-p38 MAPK, and p-ERK 1/2. Cotreatment with DJ526 and DJ-PPD downregulated the expression of proinflammatory cytokines (*IL-1β*, *IL-6*, and *TNF-α*), LPS receptor (toll-like receptor-4, *TLR-4*), proinflammatory mediators (nitric oxide and PGE_2_), and arachidonic acid pathway critical enzyme (*COX-2*). These findings demonstrate the synergistic potential anti-inflammatory activities of resveratrol- and PPD-enriched rice seed extract.

## 1. Introduction

Inflammation is a biological response to harmful pathogens, microorganisms, stimuli, or infectious agents [1]. The activation of the mitogen-activated protein kinase (MAPK) and nuclear factor kappa B (NF-κB) pathways stimulates immune protection against pathogens [2]. The activation of these pathways causes the production of proinflammatory cytokines and mediators to eliminate infections [3]. Macrophages play an important role in establishing a controllable inflammatory response [4]. During an infection, macrophages produce proinflammatory cytokines, such as IL-6, IL-1β, and TNF-α, to promote B-cell differentiation [5] and are involved in cellular activities, such as cell apoptosis, cell differentiation, and cell proliferation [6]. The excess production of proinflammatory cytokines is associated with various diseases such as type 2 diabetes [7] and chronic inflammatory lung diseases [8]. Therefore, identifying natural agents to reduce inflammatory responses is important in developing new therapeutic approaches for such diseases [9,10].

The anti-inflammatory effect of resveratrol and protopanaxadiol (PPD) has been demonstrated in several studies. Resveratrol inhibits NF-κB signaling pathway activation, which has been shown to reduce IL-1α, IL-6, and TNF-α production in the cell culture supernatant of human T-cell leukemia virus-infected cells [11]. Treatment with resveratrol markedly decreased the expression of PGE_2_, COX-2, IL-1β, IL-8, and TNF-α in lipopolysaccharide (LPS)-stimulated BV-2 cells [12,13]. Resveratrol also suppressed the mRNA and protein expression of iNOS in intestinal cells (Caco-2 and SW480) following LPS stimulation of cells and decreased nitric oxide (NO) production [14]. Resveratrol downregulates the expression of iNOS and IL-6 via inhibiting the activation of NF-κB and JAK/STAT signaling pathways in LPS-treated RAW264.7 cells [15]. In addition, the inhibition of the arachidonic acid pathway related in the enhancement of anti-inflammatory activity [16,17]. Resveratrol was shown to inhibit the arachidonic acid pathway by decreasing PGD_2_ and PGE_2_ production and suppress the COX-2 expression level in the colon of rats [18,19] and inhibit neuronal TLR and COX-2 signaling pathways [20]. PPD (20(S)-PPD-type saponin) exhibited anti-inflammatory activities by inhibiting the LPS-induced NO production in RAW264.7 cells [21]. PPD from red ginseng (*Panax ginseng* C.A. Meyer) was reported to have anti-inflammatory effects by inhibiting the production of LPS-induced NO, expression of proinflammatory genes (IL-1β and monocyte chemotactic protein-1), and activity of the arachidonic acid pathway critical enzyme (COX-2) in RAW264.7 cells [22]. The inflammatory injury in murine colitis was significantly reduced with PPD (20(S)-PPD saponin) treatment via the reduction in TNF-α, IL-1β, and IL-6 expression [23].

Our previous studies show that DJ526 significantly suppressed the activation of NF-κB (p-NF-κB p65) and MAPK (p-ERK 1/2 and p-p38 MAPK) signaling pathways, downregulating the expression of proinflammatory-related mediators [24]. The effect of DJ-PPD in LPS-stimulated RAW264.7 cells [25,26] demonstrated an anti-inflammatory potential by inhibiting the activation of NF-κB and MAPK signaling pathways. In this study, we used extracts prepared from resveratrol-enriched rice (DJ526) and PPD-producing transgenic rice (DJ-PPD) [27,28] to assess their co-anti-inflammatory effects on LPS-stimulated murine RAW264.7 cells.

## 2. Results

### 2.1. Effect of Resveratrol- and PPD-Enriched Rice Extract on RAW264.7 Cell Viability

**Figure 1:**
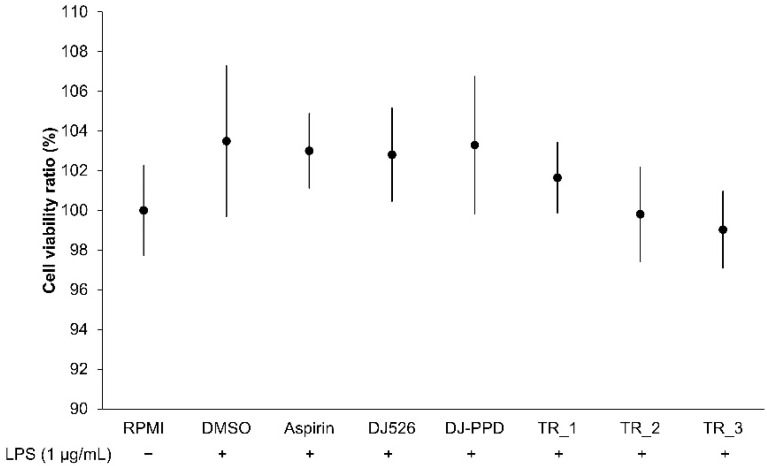
Effect of resveratrol- and PPD-enriched rice seed extracts on the viability of LPS-stimulated RAW264.7 cells. The concentrations of DMSO, aspirin, and treatments (rice seed extract and mixture) were 0.1%, 200 ng/mL, and 100 µg/mL, respectively.

Treatments were evaluated for their effect on cell viability in LPS-stimulated RAW264.7 cells. The treatments were added to cells and then stimulated with 1 µg/mL of LPS. Figure 1 shows that treatments with 0.1% of DMSO, 200 ng/mL of aspirin, 100 µg/mL of DJ526, 100 µg/mL of DJ-PPD, 100 µg/mL of TR_1 (30% DJ526 and 70% DJ-PPD), 100 µg/mL of TR_2 (50% DJ526 and 50% DJ-PPD), and 100 µg/mL of TR_3 (70% DJ526 and 30% DJ-PPD) were noncytotoxic on LPS-stimulated RAW264.7 cells (*p* < 0.05). Therefore, all treatments were used for subsequent experiments in LPS-stimulated RAW264.7 cells.

### 2.2. Effect of Resveratrol- and PPD-Enriched Rice Extract on NO Production

**Figure 2:**
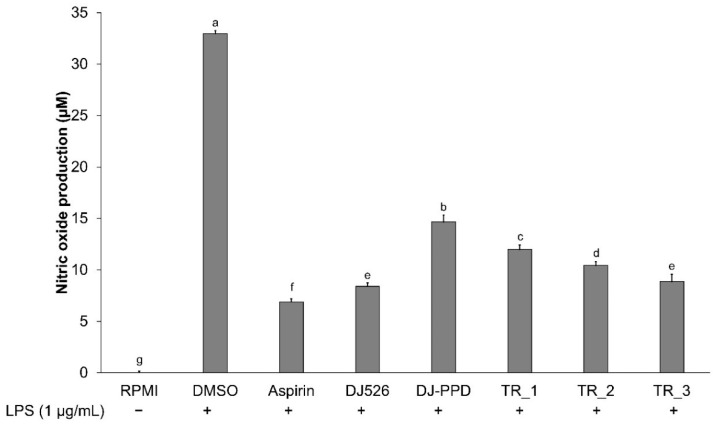
Effect of resveratrol- and PPD-enriched rice seed extracts (DJ526 and DJ-PPD, respectively) on the NO production in LPS-stimulated RAW264.7 cells. The concentrations of DMSO, aspirin, and treatments (rice seed extract and mixture) were 0.1%, 200 ng/mL, and 100 µg/mL, respectively. The significant differences in NO production are presented as lowercase letters at *p* < 0.05.

NO production in cultured media was measured using Griess reagent. Figure 2 shows that the production of NO was significantly higher in LPS-stimulated cells (DMSO group) (*p* < 0.05) than in the nontreated group (RPMI). However, the NO production in cell-cultured media significantly decreased in cells pretreated with aspirin, DJ526, DJ-PPD, TR_1, TR_2, and TR_3. As a positive control, aspirin at 200 ng/mL exhibited the highest inhibition of LPS-induced NO production in RAW264.7 cells among all treatments. The DJ526 treatment caused the highest inhibition of NO production among the treatments (74.49 ± 1.00% inhibition). For DJ-PPD, the LPS-induced NO production was significantly lower than that in the DMSO group (55.52 ± 2.04% reduction). Interestingly, the addition of DJ526 in the DJ-PPD treatment (TR_1 and TR_2) significantly reduced NO production in LPS-stimulated RAW264.7 cells when compared with that in cells treated with DJ-PPD alone. The TR_1 treatment markedly suppressed the LPS-induced NO production compared with that of DJ-PPD alone (18.22 ± 2.80% suppression). In addition, TR_2 markedly reduced NO production in LPS-stimulated RAW264.7 cells compared with that of DJ-PPD alone (28.77 ± 2.51% reduction). TR_3 treatment induced a similar inhibition of NO production compared with that after treatment with DJ526 alone. These results indicated that the addition of DJ526 in the DJ-PPD treatment significantly promoted LPS-induced NO production inhibition (*p* < 0.05).

### 2.3. Effect of Resveratrol- and PPD-Enriched Rice Extract on Inflammatory Cytokine Expression

**Figure 3:**
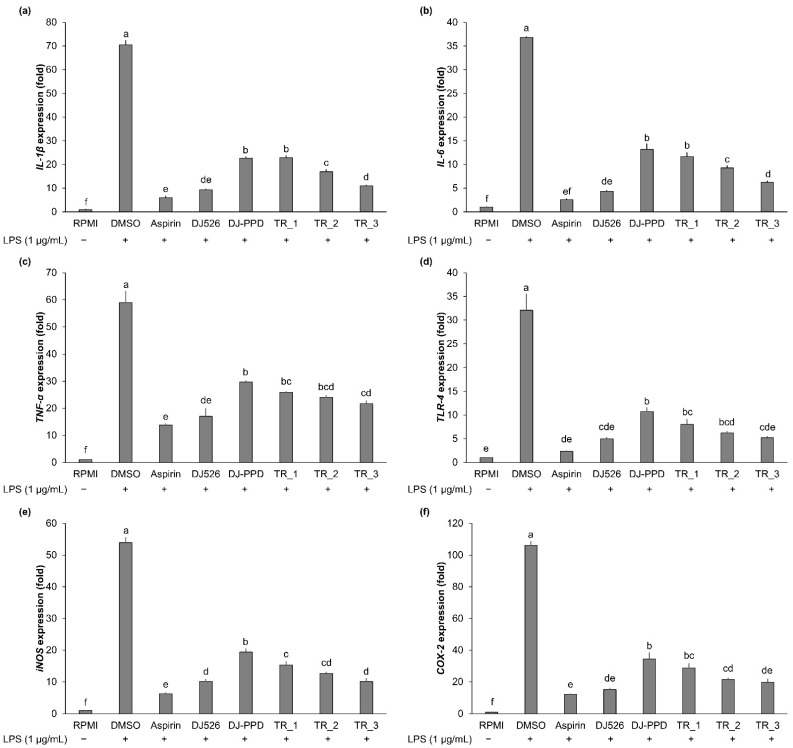
Effect of resveratrol- and PPD-enriched rice seed extracts (DJ526 and DJ-PPD, respectively) on the immune-related gene expression in LPS-stimulated RAW26.7 cells. The effect on (**a**) *IL-1β*, (**b**) *IL-6*, (**c**) *TNF-α*, (**d**) *TLR-4*, (**e**) *iNOS*, and (**f**) *COX-2* expression. The concentrations of DMSO, aspirin, and treatments (rice seed extract and mixture) were 0.1%, 200 ng/mL, and 100 µg/mL, respectively. The significant differences in gene expression levels are presented as lowercase letters at *p* < 0.05.

The expression level of immune-related genes was evaluated from cells stimulated with LPS for 6 h. The expression of these genes was upregulated in the LPS-stimulated RAW264.7 cells (DMSO group: Figure 3), whereas the expression of *IL-1β*, *IL-6*, *TNF-α*, *TLR-4*, *iNOS*, and *COX-2* was downregulated in the rice-extract-treated cells. The DJ526 treatment at 100 µg/mL exhibited the highest suppression effect of immune-related genes among the treatments, which was similar to expression levels in aspirin-treated cells (positive control group). In comparison to the effect of the DJ526 treatment, treatment with TR_3 significantly decreased the expression of LPS-induced immune-related genes to the same levels as that of the DJ526 treatment. The TR_2 treatment markedly downregulated the expression of *TNF-α*, *TLR-4*, *iNOS*, and *COX-2* to the same levels as that in the DJ526-treated cells. Moreover, the addition of 30% (*w*/*w*) of DJ526 in TR_1 significantly promoted the inhibited expression of LPS-induced immune-related genes to the same levels as found with DJ-PPD alone. These results indicate that the mixture of DJ526 and DJ-PPD significantly enhanced the inhibition of the LPS-induced immune-related gene expression.

### 2.4. Effect of Resveratrol- and PPD-Enriched Rice Extract on PGE_2_ Production

**Figure 4:**
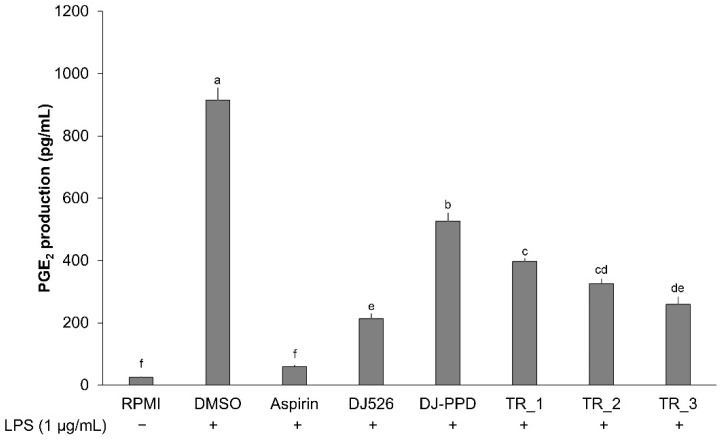
Effect of resveratrol- and PPD-enriched rice seed extracts (DJ526 and DJ-PPD, respectively) on PGE_2_ production in LPS-stimulated RAW264.7 cells. The concentrations of DMSO, aspirin, and treatments (rice seed extract and mixture) were 0.1%, 200 ng/mL, and 100 µg/mL, respectively. The significant differences in PGE_2_ production are presented as lowercase letters at *p* < 0.05.

The production of PGE_2_ was increased when cells were stimulated with 1 µg/mL of LPS (Figure 4). The treatment of cells with rice seed extract significantly suppressed LPS-induced PGE_2_ production in comparison with that in cells treated with DMSO. The positive control (aspirin) inhibited PGE_2_ production to the same level as that in the negative control (RPMI)-treated cells. Treatment with DJ526 significantly decreased the inhibited production of PGE_2_ compared with that following treatment with DJ-PPD. In addition, treatment with TR_1, TR_2, and TR_3 significantly suppressed the production of PGE_2_ compared with that in the DJ-PPD group. Among the mixture samples, the highest inhibitory effect on PGE_2_ production was observed in the TR_3 group. These results indicate that the inhibitory effect on PGE_2_ production was correlated with increasing the DJ526 ratio in the DJ526/DJ-PPD mixture (*r* = −0.9883; Pearson’s correlation coefficient; degree of freedom at 10).

### 2.5. Effect of Resveratrol- and PPD-Enriched Rice Extract on Inflammatory Pathway Signaling

**Figure 5:**
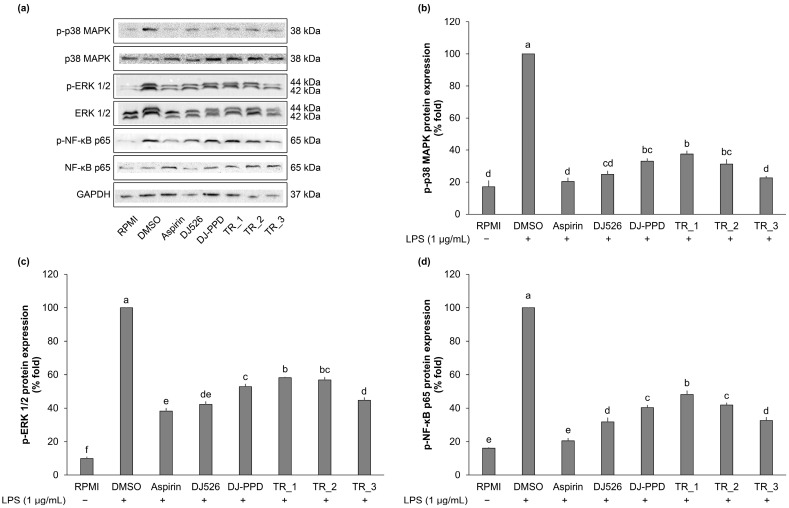
Effect of resveratrol- and PPD-enriched rice seed extracts (DJ526 and DJ-PPD, respectively) on the expression of immune-associated proteins in LPS-stimulated RAW264.7 cells. (**a**) Representative of blot analysis, (**b**) densitometric analyses of p-p38 MAPK protein expression, (**c**) densitometric analyses of p-ERK 1/2 protein expression, and (**d**) densitometric analyses of p-NF-κB p65 protein expression. The concentrations of DMSO, aspirin, and treatments (rice seed extract and mixture) were 0.1%, 200 ng/mL, and 100 µg/mL, respectively. The significant differences in protein expression are presented as lowercase letters at *p* < 0.05.

The highest expression of p-p38 MAPK, p-ERK 1/2, and p-NF-κB p65 was observed in DMSO-treated cells. Compared with that in DMSO-treated cells, the pretreatment with DJ526 and DJ-PPD and their mixture significantly inhibited the expression level of p-p38 MAPK, p-ERK 1/2, and p-NF-κB p65 (Figure 5). DJ526 produced the highest suppression of the expression of proteins associated with MAPK and NF-κB signaling pathways. Treatment with the mixture of DJ526 and DJ-PPD significantly decreased the expression of proteins associated with MAPK and NF-κB signaling pathways dose-dependently with the DJ526 ratio. Similar inhibition activities on p-p38 MAPK, p-ERK 1/2, and p-NF-κB p65 expression were observed in the TR_3 and DJ526 groups.

## 3. Discussion

LPS stimulation is frequently used to induce an in vitro inflammatory condition that mimics those in vivo [29,30,31,32,33]. LPS activates the NF-κB and MAPK signaling pathways via TLR-4 [34,35,36], an LPS receptor [37]. These pathways, when activated, generally result in an increase in the expression of known proinflammatory indicators, namely NO, iNOS, COX-2, IL-1β, IL-6, and TNF-α [38,39,40,41,42]. In addition, MAPK pathway also positively stimulates the production of prostaglandins and PGE_2_ [43,44]. The results of the present study showed synergistic anti-inflammatory regulation activity of resveratrol- and PPD-enriched rice seed extract against LPS-induced inflammatory conditions in RAW264.7 cells via the inhibition of the NF-κB and MAPK signaling pathways. Treatment of cells with TR_1-, TR_2-, and TR_3 downregulated the expression of the biomarkers p-p38 MAPK, p-ERK 1/2, and p-NF-κB p65 specifically with the DJ526 component in a dose-dependent manner (*r* = −0.9703; Pearson’s correlation coefficient). This inhibition reduced the proinflammatory biomarker content. The addition of DJ526 at 30%, 50%, and 70% (*w*/*w*) with DJ-PPD significantly inhibited the production of NO and PGE_2_ compared with that found after treatment with DJ-PPD alone (Figure 2 and Figure 4). In addition, the combination of 70% (*w*/*w*) of DJ526 and 30% (*w*/*w*) of DJ-PPD significantly inhibited the LPS-induced NO and PGE_2_ production at the same level as DJ526 treatment alone. The inhibitory effect on NO (*r* = −0.9479) and PGE_2_ (*r* = −0.9883) production correlated with the increase in the ratio of DJ526 in the DJ526/DJ-PPD mixture (Pearson’s correlation coefficient). Furthermore, treatment with DJ526 and DJ-PPD enhanced the inhibitory effect of the proinflammatory cytokines compared with that found with DJ-PPD treatment alone (Figure 3). The increasing amount of the DJ526 component in the DJ526/DJ-PPD mixture correlated with the level of inhibited expression of *IL-1β* (*r* = −0.9956), *IL-6* (*r* = −0.9838), *iNOS* (*r* = −0.9645), *COX-2* (*r* = −0.8849), *TNF-α* (*r* = −0.9553), and *TLR-4* (*r* = −0.9204).

Our results are supported by those reported by Huangfu, et al. [45], who generated resveratrol and 20(S)-protopanaxadiol complex nanoparticles (RES@PPD, NPs) to investigate their anti-inflammatory and antioxidant activities. The treatment with NPs significantly suppressed the M1 polarization of macrophages, which are related to the secretion of proinflammatory cytokines [46], and promoted M2 polarization of macrophages, which are related to the inflammatory resolution or anti-inflammatory functions [47]. In addition, the NPs exhibited anti-inflammatory activities in the male Sprague–Dawley rat model of periodontitis by suppressing the expression of proinflammatory cytokines and increasing that of anti-inflammatory cytokines. Several studies have demonstrated the anti-inflammatory effects of resveratrol together with other compounds. Resveratrol combined with vitamin E significantly inhibited the phosphorylation of NF-κB p65, which then decreased the content of inflammatory mediators, such as NO, IL-1β, IL-6, TNF-α, and TLR-4 [48]. The combination of naproxen and resveratrol markedly promoted the anti-inflammatory activities by inhibiting the production of NO and inflammatory cytokines, such as TNF-α, COX-2, iNOS, and IL-6 by inhibiting the NF-κB, MAPK, and phosphoinositide-3 kinase (PI3K)/protein kinase B (Akt) signaling pathways [49]. The synergic effect of PPD has also been shown in several studies. Wang, et al. [50] reported that PPD effectively increased the effects of chemotherapy with 5-FU in patients with colorectal cancer by inhibiting the proliferation of HCT-116 human colorectal cancer cells when compared with the treatment of PPD or 5-FU alone. Ben-Eltriki, et al. [51] evaluated the combined effect of PPD and 1,25-dihydroxyvitamin D3 (calcitriol) and showed that treatment with PPD and calcitriol in combination significantly inhibited tumor growth in an androgen-independent C4-2 xenograft CRPC mouse model. The combination of PPD and cannabidiol (CBD-PPD coloading liposome, CP-liposomes) effectively inhibited the growth of the 4T1 breast cancer cell line more than that with CBD or PPD treatment alone [52]. In addition, administering resveratrol and *Ligilactobacillus salivarius* Li01 in combination significantly reduced the production of proinflammatory cytokines (*IL-1β* and *IL-6*) and increased that of an anti-inflammatory cytokine (*IL-17A*) in mouse serum [53].

## 4. Materials and Methods

### 4.1. Treatment Preparation

Treatments were prepared by DJ526 with DJ-PPD at various ratios (Table 1). DJ526 and DJ-PPD extracts are the same extracts as previously used [24,25]. DJ526 contained 4.724 and 2.605 ug/g dry weight of piceid and resveratrol, respectively [24], while DJ-PPD contained 7.28 ± 0.64 µg/g dry weight of protopanaxadiol [25]. After mixing, the treatments were prepared at 100 mg/mL in DMSO.

### 4.2. Cell Viability and NO Production

**Figure 6:**
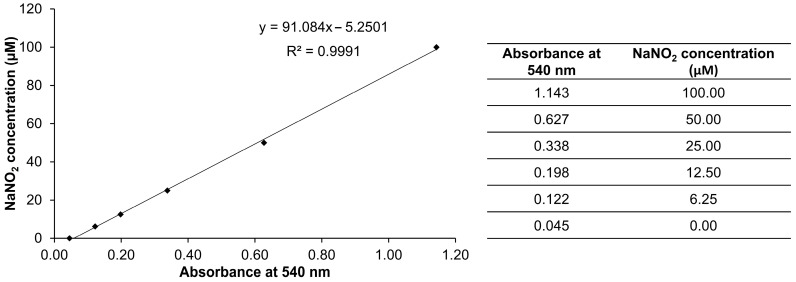
The calibration curve of NaNO_2_ (0.00–100.00 µM).

Cells in RPMI-1640 media (10% fetal bovine serum and 1% penicillin/streptomycin) at the concentration of 1 × 10^5^ cells were placed in a 96-well plate and the plate was incubated at 37 °C and 5% CO_2_ for 24 h. The cultured medium was discarded and the treatments (0.1% of DMSO, 200 ng/mL of aspirin, and 100 µg/mL of DJ526, DJ-PPD, TR_1, TR_2, and TR_3: 100 µL) were added into the specific wells. Aspirin at the concentration of 200 ng/mL served as a positive control. Aspirin, one of the most important members of nonsteroidal anti-inflammatory drugs, was used in treatment of inflammation [54]. Aspirin inhibits cyclooxygenase (COX), prostaglandins biosynthesis, and thromboxane biosynthesis [55]. Several studies demonstrated that aspirin is involved in anti-inflammatory processes through inhibiting the NF-κB and MAPK pathway [56,57,58], which led to the downregulation of NO, COX-2, PGE_2_, and proinflammatory cytokines [59,60,61]. After 1 h incubation, cells were stimulated with or without 1 µg/mL of LPS (Sigma-Aldrich, St. Louis, MO, USA). After 24 h stimulation, the cultured medium and stimulated cells were collected to assay NO production and cell viability. Culture media (100 µL) was transferred to a new 96-well plate and then mixed with a 100 µL working solution of Griess reagent (Sigma-Aldrich, St. Louis, MO, USA). The mixture was incubated for 15 min at room temperature in the dark and then the absorbance of the solution was measured at 540 nm. A standard curve of sodium nitrite was constructed for quantification of NO production (Figure 6). An EZ-Cytox Cell Viability Assay Kit (DoGenBio, Seoul, Republic of Korea) and stimulated cells were used for measuring the cytotoxicity of treatments. A working solution of an EZ-Cytox Cell Viability Assay Kit (10-fold dilution in 1× PBS: 110 µL) was added to stimulated cells, which were then incubated at 37 °C for 4 h. The solution (100 µL) was transferred to a new 96-well plate and the absorbance was measured at 450 nm. Cell viability was calculated by the following equation:

(1) $$\mathrm{Cell}\;\mathrm{viability}\;\mathrm{ratio}\;(\%)=\frac{\mathrm{An}\;\mathrm{absorbance}\;\mathrm{at}\;450\;\mathrm{nm}\;\mathrm{of}\;\mathrm{treatment}\;\mathrm{group}}{\mathrm{An}\;\mathrm{absorbance}\;\mathrm{at}\;450\;\mathrm{nm}\;\mathrm{of}\;\mathrm{control}\;\mathrm{group}}\;\times \;100,$$

where control represents the untreated group.

The NO production in culture media from each treatment was calculated according to Equation (2).

(2) Nitric oxide production (µM)=91.084x-5.2501, 

where x represents the absorbance value at 540 nm.

### 4.3. RNA Extraction

Total RNA was extracted from each treatment group using a method described in the previous study [26]. Briefly, the treated cells were washed with ice-cold 1× PBS, lysed with TRI reagent™ (Invitrogen, Waltham, MA, USA), and then mixed with 200 µL of chloroform. After centrifugation, the upper-phase solution was transferred to the new tube. Total RNA was precipitated with isopropanol and the RNA pellet was washed with 70% EtOH. The RNA pellet was dissolved in nuclease-free water and kept at −80° until use.

### 4.4. RNA Quantification and cDNA Synthesis

The RNA concentration and RNA quality were evaluated using a SpectraMax^®^ ABS Plus Microplate Reader (Molecular Device, San Jose, CA, USA) with a previously described method [62]. The RNA quality was shown as the ratio of A260:A280 and A260:A230 (acceptation range is 1.800–2.000; Table 2). The total RNA at 1 µg was used for the cDNA synthesis using a Power cDNA Synthesis Kit (Intron Biotechnology, Seongnam-si, Republic of Korea). The working cDNA solution was prepared at 5 ng/µL in nuclease-free water.

### 4.5. Inflammatory-Related Gene Expression Measurement

The mRNA expression levels were measured using a CFX Connect Real Time System (Bio-Rad, Hercules, CA, USA) in the total 20 µL of RealMOD™ Green W^2^ 2 × qPCR mix (Intron Biotechnology). Specific primer sequences used were described in the previous study [62]. The PCR conditions are shown in Table 3.

### 4.6. PGE_2_ Production Assay

**Figure 7:**
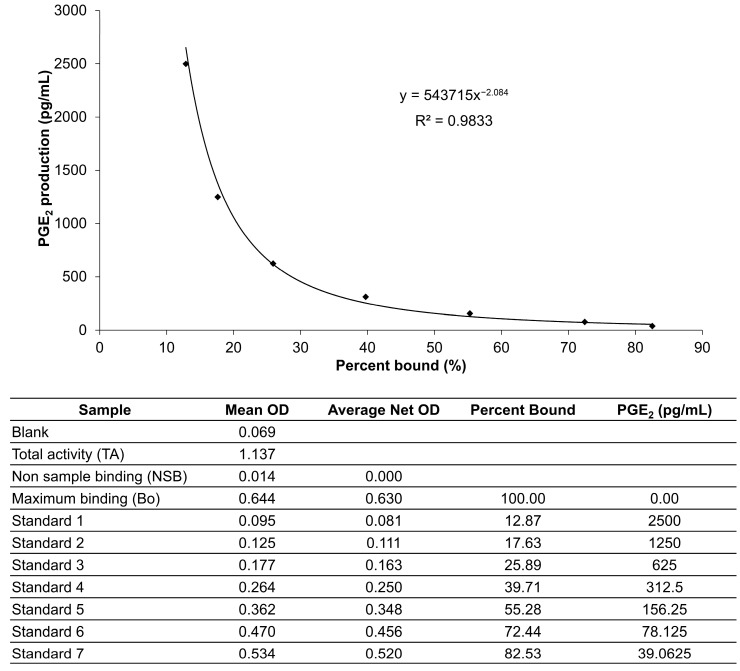
Standard curve of PGE_2_ production over the range of 39.0625–2500 pg. OD = optical density.

The cultured medium was collected and centrifuged at 3000 rpm for 10 min. The supernatant was used to determine the production of PGE_2_ with a PGE_2_ ELISA kit (Enzo Life Science, Farmingdale, NY, USA), according to the manufacturer’s instructions. Sample and standard solution (100 µL/well) were added into appropriate wells. Assay buffer was added into the nonsample binding (NSB: 150 µL/well) and maximum binding (Bo 100 µL/well) wells. The conjugate buffer (50 µL/well) was then added to each well, except for the total activity (TA) and blank wells, and then 50 µL of antibody was added per well, except for the blank, TA, and NSB wells. The plate was incubated at room temperature for 2 h at 500 rpm. Culture media were discarded and wells were washed with wash solution three times. After washing, the solution in the TA wells was mixed with conjugate solution (5 µL/well). The pNpp substrate solution (200 µL/well) was pipetted to each well and the plate was incubated at room temperature for 45 min. Finally, the stop solution (5 µL/well) was added per well and the absorbance was measured at 405 nm immediately. The PGE_2_ standard was generated by plotting the graph of percent bound versus concentration of PGE_2_ (Figure 7). The PGE_2_ production in the cultured medium was calculated according to Formula (3).

(3) $${\mathrm{PGE}}_{2}\;\mathrm{production}\;(\mathrm{pg}/\mathrm{mL})\;=\;{543715x}^{-2.084},$$

where x represents the percent bound value.

### 4.7. Western Blot Analysis

The radioimmunoprecipitation assay buffer (Geneall Biotechnology, Seoul, Republic of Korea) was used for protein extraction from each treated culture. Cells were harvested using trypsin-EDTA and centrifugation at 13,000 rpm and 4 °C for 1 min. RIPA buffer supplemented with 1× Protease Inhibitor Cocktail Kit 5 (Bio-Medical Science Co., Ltd., Seoul, Republic of Korea) was added to the cell pellet. The solution was incubated on ice for 3 min and then centrifuged at 13,000 rpm and 4 °C for 30 min. The protein concentration in the resultant supernatant was measured using Bradford reagent (Sigma-Aldrich) by comparison with a bovine serum albumin standard curve. Protein sample (30 µg) from each treatment was separated in sodium dodecyl–sulfate polyacrylamide gel. The separated protein was transferred onto a nitrocellulose membrane (Immobilon^®^-P Transfer Membrane, Merck, Millipore, Burlington, MA, USA). The membrane was washed with 1× tris-buffered saline containing 0.1% Tween^®^ 20 detergent (TBST) and was then incubated with 5% (*w*/*v*) skim milk in 1× TBST (blocking solution) at room temperature for 2 h. The membrane was incubated with antibodies in blocking solution at the ratio 1:1000 (*v*/*v*) specific for p38 MAPK (Santa Cruz Biotechnology, Dallas, TX, USA), NF-κB p65 (Santa Cruz Biotechnology), and ERK 1/2 (Santa Cruz Biotechnology). Membranes were incubated with antibodies in blocking solution at the ratio 1:2000 (*v*/*v*) specific for phosphorylation (p)-p38 MAPK (Cell Signaling Technology, Danvers, MA, USA), p-NF-κB p65 (Cell Signaling Technology), and p-ERK 1/2 (Cell Signaling Technology). Glyceraldehyde-3-phosphate dehydrogenase (GADPH, Santa Cruz Biotechnology) was used as the reference protein at 1:5000 (*v*/*v*) in TBST. Membranes were incubated with primary antibody at 4 °C overnight with mild agitation and then washed several times with 1× TBST at room temperature with strong agitation. Membranes were then incubated in 1:5000 (*v*/*v*: secondary antibody–blocking solution) of the secondary antibody (goat antirabbit IgG (H + L)-horseradish peroxidase (GenDEPOT, Barker, TX, USA) or m-IgG^®^ BP-horseradish peroxidase (Santa Cruz Biotechnology)) at room temperature for 2 h. Membranes were incubated with Pierce ECL plus Western blotting substrate (Thermo Scientific™, Waltham, MA, USA) at room temperature for 10 min in the dark. Signals were captured, recorded, and the band intensity quantified using ChemiDoc Image System (Bio-Rad) and Image Lab Software (version 6, Bio-Rad).

### 4.8. Statistical Analysis

All calculations were performed using Statistix 8.1 (Statistix, Tallahassee, FL, USA). Treatment group means were compared by one-way analysis of variance followed by post hoc Duncan’s multiple range tests. A *p*-value < 0.05 was considered significant for all tests.

## 5. Conclusions

This study demonstrated the co-ordination anti-inflammatory effect of resveratrol- and PPD-enriched rice seed extracts on LPS-stimulated RAW264.7 cells. Treatment with DJ526 and DJ-PPD in combination significantly inhibited the MAPK and NF-κB signaling pathways. The cotreatment with DJ526 and DJ-PPD downregulated the production of NO and PGE_2_ and the expression of immune-associated genes in LPS-stimulated RAW264.7 cells. Therefore, we suggest that extracts of resveratrol- and PPD-enriched rice in a combination could be used as an anti-inflammatory agent.

## Figures and Tables

**Table 1:** Treatment preparation used in this study.

Treatment	DJ526 (% *w*/*w*)	DJ-PPD (% *w*/*w*)	Piceid Content (×10^−2^ ng)	Resveratrol Content (×10^−2^ ng)	PPD Content (×10^−2^ ng)
DJ526	100	0	47.2	26.1	0.0
DJ-PPD	0	100	0.0	0.0	72.8
TR_1	30	70	14.2	7.8	51.0
TR_2	50	50	23.6	13.1	36.4
TR_3	70	30	33.0	18.3	21.8

**Table 2:** The quantity and quality of total RNA.

Treatment	A260:A280	A260:A230	Concentration (ng/µL)	SD *	CV **
Nontreatment (RPMI)	1.974	1.851	351.49	8.12	2.31
DMSO	1.996	1.832	389.97	12.88	3.30
Aspirin	1.993	1.942	802.40	29.99	3.74
DJ526	2.001	1.905	826.93	17.33	2.10
DJ-PPD	1.989	1.988	885.23	30.78	3.48
TR_1	1.969	1.841	827.03	11.24	1.36
TR_2	1.997	1.915	836.19	17.22	2.06
TR_3	1.975	1.887	958.79	12.05	1.26

* Standard deviation (SD). ** Coefficient of variation (CV).

**Table 3:** PCR reaction conditions used in this experiment.

Process	Temperature (°C)	Time	Number of Cycles
Predenaturation	95	10 min	1 cycle
Denaturation	95	20 s	40 cycles
Annealing	60	20 s	
Elongation	72	30 s	
Final elongation	72	5 min	1 cycle

## Data Availability

All the applicable data have been provided in the manuscript. The authors will provide additional details if necessary.
